# Team science competencies for clinical research professionals: A multileveled Delphi approach

**DOI:** 10.1017/cts.2024.509

**Published:** 2024-04-01

**Authors:** Angela Mendell, Jessica Fritter, Shirley Helm, Bernadette Capili, Laura Hildreth, Kathryn Johnson, Christa Varnadoe, Elizabeth Kopras, Jen Sprecher, Nicole Summerside, Karen Carter, Andrea Ronning, Nicole Exe, H. Robert Kolb, Carolynn T. Jones

**Affiliations:** 1 University of Cincinnati, Cincinnati, OH, USA; 2 The Ohio State University, Columbus, OH, USA; 3 Virginia Commonwealth University, Richmond, VA, USA; 4 Rockefeller University, New York, NY, USA; 5 Icahn School of Medicine at Mount Sinai, New York, NY, USA; 6 University of Vermont, Burlington, VT, USA; 7 University of Washington, Seattle, WA, USA; 8 University of Michigan, Ann Arbor, MI, USA; 9 University of Florida, Gainesville, FL, USA

**Keywords:** Clinical research professional, clinical research competencies, interdisciplinary teams, professional development, team science competencies

## Abstract

**Background:**

The knowledge, skills, and abilities needed for clinical research professionals (CRPs) are described in the Joint Task Force (JTF) for Clinical Trial Competencies Framework as a basis for leveled educational programs, training curricula, and certification. There is a paucity of literature addressing team science competencies tailored to CRPs. Gaps in training, research, and education can restrict their capability to effectively contribute to team science.

**Materials/Methods:**

The CRP Team Science team consisted of 18 members from 7 clinical and translational science awarded institutions. We employed a multi-stage, modified Delphi approach to define “Smart Skills” and leveled team science skills examples using individual and team science competencies identified by Lotrecchiano et al.

**Results:**

Overall, 59 team science Smart Skills were identified resulting in 177 skills examples across three levels: fundamental, skilled, and advanced. Two examples of the leveled skillsets for individual and team competencies are illustrated. Two vignettes were created to illustrate application for training.

**Discussion:**

This work provides a first-ever application of team science for CRPs by defining specific individual and team science competencies for each level of the CRP career life course. This work will enhance the JTF Domains 7 (Leadership and Professionalism) and 8 (Communication and Teamwork) which are often lacking in CRP training programs. The supplement provides a full set of skills and examples from this work.

**Conclusion:**

Developing team science skills for CRPs may contribute to more effective collaborations across interdisciplinary clinical research teams. These skills may also improve research outcomes and stabilize the CRP workforce.

## Introduction

Clinical research professionals (CRPs) are essential members of clinical translational science teams, representing a large heterogeneous group of professionals, including clinical research nurses, coordinators and a large cadre of diverse specialties that manage clinical research activities from inception through operation to dissemination [1]. Career pathways for CRPs can be multifaceted, with opportunities for growth and development in different areas of clinical research, such as project management, regulatory affairs, or data management, in addition to direct participant interactions as part of study coordination. CRPs work in community, outpatient, and in-patient settings to operationalize and manage clinical research studies. The knowledge, skills, and abilities (KSAs) needed for CRP role activities and progression are described in the Joint Task Force (JTF) for Clinical Trial Competencies Framework as a basis for leveled educational programs, training curricula, and certification [2,3]. Despite the crucial role of CRPs in translational science, there is a noticeable lack of published literature addressing team science competencies and training tailored for CRPs. This gap highlights the need for a more comprehensive understanding of the unique skills and expertise required by CRPs to effectively engage within the expanding web of interdisciplinary teams.

Moreover, gaps in training, research, and education for CRPs can limit their ability to engage in and contribute to team science efforts fully. Benchmarks for CRP training and certification have been derived from the JTF Competency Framework [2]. Many of these benchmarks focus on the operational competency domains: JTF Domain 2 (Ethical and Participant Safety Considerations), JTF Domain 3 (Investigational Products Development and Regulation), JTF Domain 4 (Clinical Study Operations/Good Clinical Practice), JTF Domain 5 (Study and Site Management) and JTF Domain 6 (Data Management and Informatics) [4,5]. However, there is a lack of attention, training, certification content, and published literature on leadership and professionalism, communication, and teamwork, found in JTF Domains 7 and 8 [6]. While team science competency literature is lacking there is literature on how to form CRP teams highlighted by a national pediatric clinical trials network in the Institutional Development Awards (IDeA) program [7]. Another publication, which featured a focus group exploring communication-related stressors in CRP roles and suggested that Leadership and Professionalism (JTF Domain 7) ground the activities of translational science and serve to interconnect the other competency domains, further suggested that communication and teamwork (JTF Domain 8) operate as the hub that mechanizes operations [6]. Addressing unmet needs in CRP team science skillsets training and research will enhance the professional development of CRPs and maximize the overall effectiveness of translational science teams.

The CRP workforce, especially in academic medical center research sites, is at a crisis point with unprecedented staff turnover that negatively impacts study operations and associated care of patients and study participants [8]. This current workforce crisis highlights the importance of defining CRP roles within the context of established clinical research competencies, including the establishment of competency-based job titles and progression pathways [1,2]. Another critical issue is competency-based onboarding training and continuing education [9]. Factors related to the “great resignation,” shifts in workplace settings (on-site and remote) and an increase in technology have intensified the need to strengthen the team science skills of CRPs, including supervisors and research department managers. The unique needs of the post-COVID workforce stress the importance of training staff members and managers in team science to strengthen employee engagement, thus improving the intended outcomes of the entire research enterprise [10].

The National Research Council defines team science as “scientific collaboration, i.e., research conducted by more than one individual in an interdependent fashion, including research conducted by small teams and larger groups.” (p. 22) [11] Since this publication, multiple initiatives have been initiated that are dedicated to team science and the science of team science. Some of these initiatives indicate that having diverse representation within science teams, when high functioning, can improve the quality and outcomes of the team’s goals by bringing a wide array of perspectives to bear towards reaching those goals [12–15] However, many of those efforts have been primarily focused on translational researchers, namely principal investigators and those being trained to progress to principal investigator roles [16–19] Interdisciplinary team science training for clinicians has also been implemented across multiple campuses with National Institutes of Health support [20] Team science training for these groups aims to accelerate the translation of scientific discoveries into clinical practice and improve patient care by leveraging each team member’s unique skills, knowledge, and perspectives. In clinical translational research, interdisciplinary team science involves the integration of various disciplines, such as medicine, nursing, pharmacy, epidemiology, biostatistics, and bioinformatics, among others. Efforts to generate training in team science that incorporates community researchers, community health workers, and members of the community have been spearheaded by the National Center for Advancing Translational Sciences (NCATS) [21] Community researchers play a vital role in connecting research efforts with their communities, ensuring that studies are culturally appropriate and relevant to the target population. However, there is a paucity of literature on CRP team science. Since CRPs are essential members of clinical research teams, enhancing focused team science competency training for CRPs will ultimately contribute to more effective team cohesion, collaboration, improved research operations and outcomes, and a more substantial impact on patient care and public health [22]. By fostering effective communication, collaboration, and problem-solving within these multidisciplinary teams, team science promotes innovation, enhances research efficiency, and ultimately drives healthcare and public health advancements.

A recent publication by Lotrecchiano *et al*. [23] defined core competencies for team science that are interlaced across five individual and thirteen team-related team science core competencies. Members of this team science group formed a task force to explore team science across the career lifespan using three constituency groups: faculty and trainees; (2) community researchers; and (3) CRPs [24]. The workgroups adopted the Lotrecchiano *et al*. [23] as a basis of exploring team science competencies for each segment. This paper describes the process and results of the work of the CRP team science constituency group. Our volunteer group consisted of members at medical research institutions who have received Clinical and Translational Science Awards (CTSA) program funding including CRPs and members who have roles in team science training, education and consultation at their institution. Two co-chairs of the CRP constituency group intentionally recruited a multidisciplinary team representing clinical research professionals (CRPs) in various roles and those working in the team science space. The CRP constituency group included 18 members working in seven Clinical Translational Science Award (CTSA) program sites. Of these, seven were clinical research nurses, ten were clinical research managers/administrators with study coordinating experience, including educators (academic and training), and other clinical research coordination experience (two were registered dieticians, and two were basic science research assistants who also worked in clinical research or pre-clinical research areas), and four have experience in team science. Four of the 18 members rotated off the group after six months due to competing commitments. The co-chairs met monthly in planning sessions and monthly with the full CRP constituency group via Zoom (Zoom Video Communications Inc., San Jose, CA). The team used the document-sharing and editing platform Google Drive. We applied a modified Delphi approach to expand skillsets for Lotrecchiano *et al*. [23, 25] individual and team competencies for CRPs across the career lifespan from novice to expert. The study aimed to articulate skillsets that CRPs can learn and embrace to strengthen personal and team growth to enhance efficient and effective performance across the complex overlapping sets of teams they encounter in their roles. While our team consisted of CRPs at several CTSA research institutions, we hope this informs future work in this area for CRPs working in sites that are without a CTSA award.

## Materials and methods

### Modified Delphi approach

Our work was informed by the team science competencies identifed by Lotrecchiano *et al*. [23], (Table 1) which consisted of five “individual” competencies and eight “team” competencies.

**Table 1.** Individual and team competencies by Lotrecchiano *et al* [23]

Facilitating Awareness and Exchange (Individual) Cognitive Openness and Intersubjectivity (Individual) Self-Awareness (Individual) Interdisciplinary Research Management (Individual) Passion and Perseverance (Individual) Team Roles (Team) Team-Based Communication (Team) Shared Visioning (Team) Understanding Complexity (Team) Team Learning and Adaptive Behaviors (Team) Meeting Management (Team) Interdisciplinary Collaboration (Team) Building Trust (Team)

We developed a multi-stage approach and used a modified Delphi method to define leveled team science competencies for CRPs. A Delphi approach uses a set of experts to gain consensus opinions on a particular issue, using rounds of review, reflection, and discussion to achieve consensus on a specific topic. It uses an iterative process, involving multiple rounds whereby responses are combined and shared with the group [25–27]. The Modified Delphi approach provides a structured communication approach, gives voice to individuals in workgroups and through the iterative process work is accomplished, avoiding “group think.” It is used when there is existing knowledge or theories about existing knowledge [28]. To manage the rotation of the Delphi cycles, the team was divided into four smaller discussion groups, with a volunteer team leader for each (AM, CJ, JF, SH). The discussion groups via Zoom or E-mail, which entailed successive reviews and discussions to achieve project goals. Finally, the entire group met monthly via Zoom to review the work completed by each group and discuss outputs. The outputs underwent iterative edits for each phase until group consensus was reached.

#### Stage 1- define CRPs

As a collective CRP research team, we defined that CRPs develop, demonstrate, and disseminate scientific and operationalized innovations that improve the efficiency and effectiveness of clinical translation from first-in-human studies to community health dissemination. Moreover, we recognized that CRPs were a diverse network of non-faculty individuals working in various roles in the clinical research institution. Those roles include but are not limited to clinical research coordinators (CRCs), clinical research nurses (CRNs), clinical research assistants (CRAs), data managers, regulatory affairs professionals, compliance officers, quality assurance officers, lab personnel, and pharmacy personnel.

#### Stage 2- define the CRP career life-course

Stage 2 focused on defining the life course for CRP professional progression. CRPs often come into clinical research as novices to the workforce or from other professional realms. Most CRPs were unaware that clinical research professional roles existed prior to landing their first job in clinical research [8,9]. Despite expertise in other areas (e.g., nursing, pharmacy, administration), those who enter a new role in clinical research experience a return to novice status in terms of clinical research operational skill sets. We selected the three CRP professional levels previously defined by the Joint Task Force for Clinical Trial Competence that condensed the five novice to expert stages defined by Dreyfus [29] into three stages of skill acquisition (fundamental, skilled, and advanced) that followed job title role progression [3,30].

- Fundamental: *Perform tasks and/or display knowledge at an essential level; may need assistance, coaching, or supervision.*
- Skilled: *Act independently, consistently, and accurately at a moderate level of expertise; independently identify resources and use available tools effectively*.
- Advanced: A*dvanced knowledge, skills, and abilities (KSAs), and can coach, mentor, and supervise; able to think critically and to problem-solve*.

After examining the life course and competencies by role, the group determined that the three levels and the individual and team competencies applied equally to individuals, whether lab personnel, pharmacy, CRCs, CRNs, or other defined CRP roles.

#### Stage 3- defining smart skills and leveled examples for individual and team competencies

**Figure 1.**
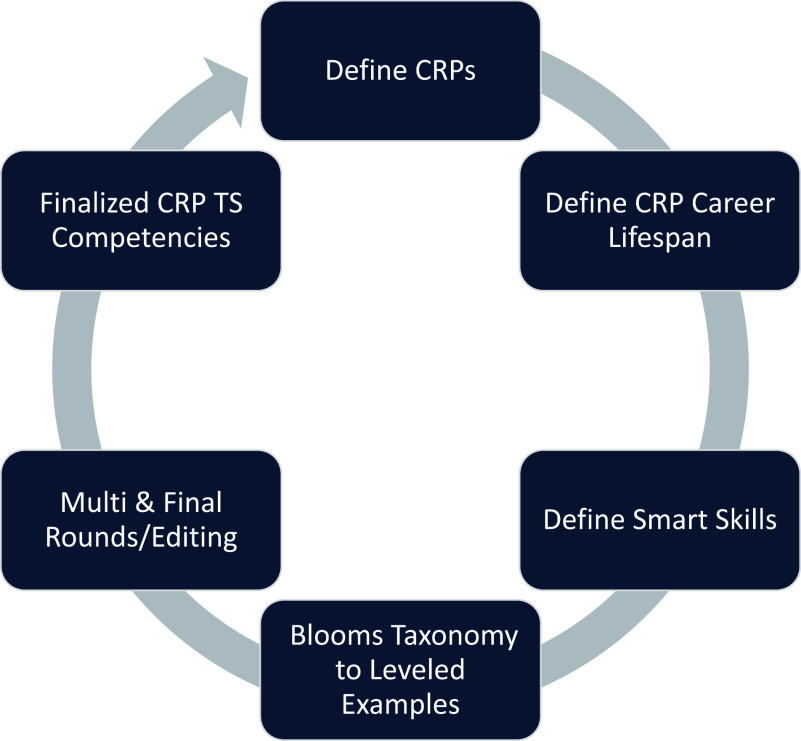
Process of defining smart skills and leveled examples.

Using an Excel Worksheet with tabs created for each of the 13 core competencies, a worksheet shell was developed to record defined smart skills and leveled skill examples generated by the four groups. Each team was responsible for reviewing and reframing examples of the CRP Team Science skills at the Fundamental, Skilled, and Advanced levels and were assigned specific individual and team competencies as outlined by Lotrecchiano *et al.* [23] (Fig. 1). The groups we assigned to identify 4 to 6 specific “smart skills” for the defined individual and team competency and define examples based on experience levels (fundamental, skilled, advanced). The initial round ensured that teams were working similarly and established reliability across raters. (See Table 2)

**Table 2.** Planned workgroup Delphi rounds per competency

Discussion Group	CRP Competency Assignments Per Round*				
	Round 1	Round 2	Round 3	Round 4	Round 5
**1**	1	5,9	13	4, 8, 12	1–13
**2**	2	6	10	3, 7, 11	1–13
**3**	3	7	11	2, 6, 19	1–13
**4**	4	8	12	1, 4, 8, 13	1–13

CRP competencies are numbered and described in Table 1.

#### Stage 4- apply Bloom’s taxonomy to leveled skills

The group determined that Bloom’s taxonomy [31] provided a good approach for creating clear, leveled, measurable competencies at ascending KSA levels. We used a consistent set of Bloom’s terms for fundamental, skilled, and advanced levels. The four discussion groups applied these in edits to their initial assigned competencies and then again in a series of group Zoom meetings.

#### Stage 5- gaining consensus: final editing rounds

The competency worksheet was periodically shared with the leaders of the team science constituency groups (Faculty/Trainee and Community Researchers) throughout the life course project. Furthermore, we presented this work at the Translational Science 2022 Conference, Association of Clinical Research Professionals, International Association of Clinical Research Nurses and Society of Clinical Research Associates to gain feedback from attendees, where the work was received positively [32–35]. Finally, we completed our rounds of editing by collectively reviewing and editing team science Smart Skills and leveled skills examples, culminating the project (See Supplement).

#### Stage 6- develop vignettes to illustrate training

After final editing, two vignettes were developed to illustrate the application of the individual and team-leveled team science competencies for CRPs. The intent was to provide a context for developing future training materials.

## Results

Fifty-nine smart skills were identified, derived from the thirteen team science competencies of Lotrecchiano *et al*. [23] Each Smart Skill illustrated leveled skills examples (*n* = 177). Table 3 illustrates two of the leveled Smart Skills and leveled examples developed for “Facilitating Awareness and Exchange” at the individual level. Table 4 illustrates two of the Smart Skills and leveled examples developed for “Team Learning and Adapting Behaviors” at the team level. The entire set of CRP Team Science Individual and Team Competencies, CRP Smart Skills and Leveled Examples are found in the article Supplement.

**Table 3.** Bloom’s taxonomy applied to a CRP smart skill examples

1. FACILITATING AWARENESS AND EXCHANGE^1^ (Individual Competency)			
Defined as: *Sharing information and perspectives, active listening and probing, reframing skills* ^*1*^			
CRP smart skill	Fundamental	Skilled	Advanced
Active listening	***Identify*** *examples of active listening during training sessions*	***Demonstrate*** *active listening to gain clarity of exchanged messages.*	***Integrate*** *active listening into staff training and meetings*
Relational openness	***Recognize*** *the importance of relational openness as team member.*	***Exhibit*** *relational openness by welcoming and introducing team members.*	***Create*** *a welcoming, inclusive, and positive environment.*

**Table 4.** Bloom’s taxonomy applied to a CRP team competency

7. TEAM-BASED COMMUNICATION (Team Competency)			
Defined as: *Sharing information and perspectives, active listening and probing, reframing skills* ^*1*^			
CRP smart skill	Fundamental	Skilled	Advanced
Team agreements	***Describe*** *team agreements*	***Demonstrate*** *team agreements and norms*	***Integrate*** *team agreements in practice*
Communication methods	***Recognize*** *various communication methods and team preferences*	***Exhibit*** *preferred team communication methods*	***Construct*** *team communication methods for process improvement*

### Applying CRP team competencies in training vignettes

**Figure 2.**
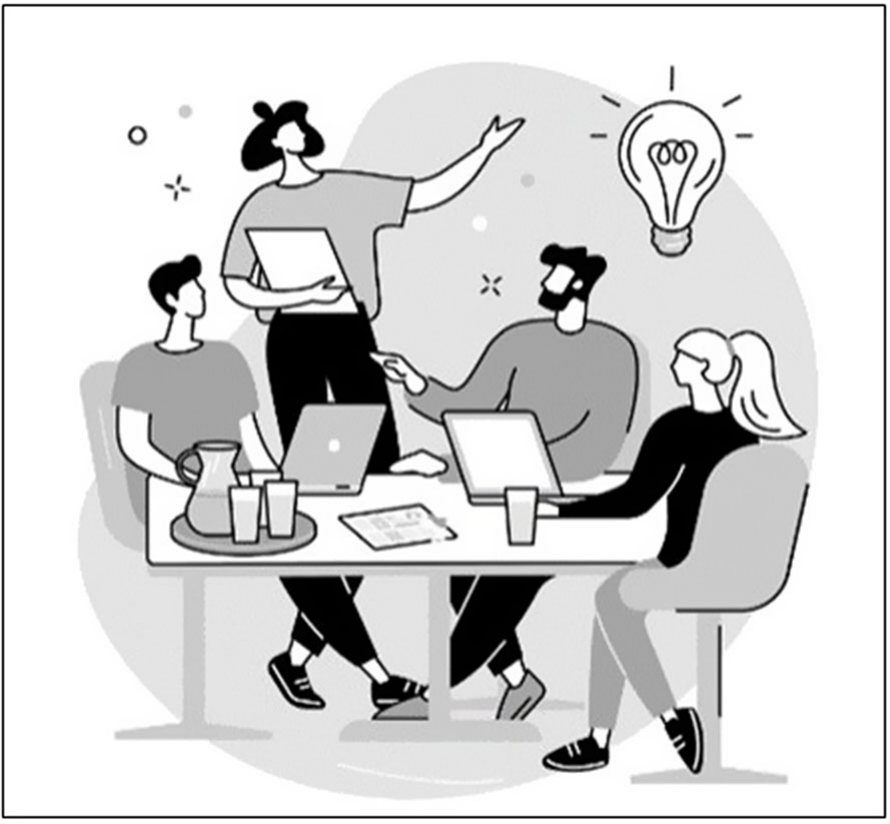
Vignette 1: sample individual competency *[Image: stock.adobe.com/visual generation].*

**Figure 3.**
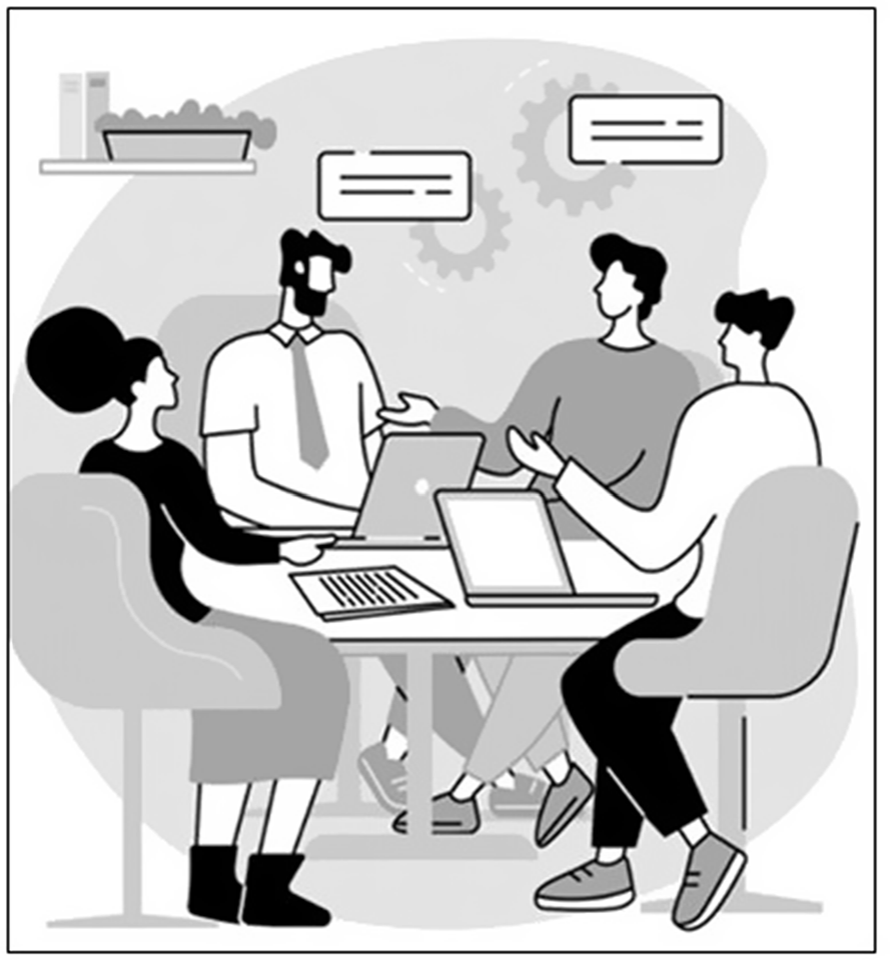
Vignette 2: sample team competency *[Image: stock.adobe.com/visual generation]*.

We developed two vignettes to provide relevant, realistic, and applicable examples of applying the CRP individual and team competencies to illustrate day to day team activities of CRPs in their roles. The vignettes highlight an example of how to implement measurable SMART skills at the fundamental, skilled, and advanced levels when applied to individual and team CRP Team Science Competencies. The two vignettes and associated tables (Fig. 2, Table 5 and Fig. 3, Table 6) follow a Quality Assurance Officer (a CRP) who is tasked with monitoring, reviewing, and training staff members on informed consent processes to ensure that (a) the participant’s rights, safety, and welfare are protected, (b) informed consent is conducted in accordance with the approved research plan, and (c) it complies with all applicable federal regulations and institutional policies.

**Table 5.** Vignette 1: the quality assurance (QA) officer supports “facilitating awareness and exchange” and implements leveled “open sharing”

Fundamental	Skilled	Advanced
***Explain*** *the benefits of openness in sharing*	***Practice*** *openness in sharing skills with others*	***Mentor*** *openness and cross-team sharing*
The QA officer understands the benefits of openness in sharing. They explain to others how open sharing supports Good Clinical Practice throughout the informed consent process by reducing the risk of errors in obtaining and documenting informed consent of research participants. They ask group attendees to give examples of how this is put into practice. One example given was using plain language to describe a risk factor.	The QA officer openly shares their skills with their research colleagues ensuring they are comfortable and confident with the expectations of their roles and responsibilities in maintaining real-time quality performance. The team knows their role is to evaluate the informed consent process for good source documentation, completion, and accuracy. Without hesitation, they approach their colleagues to resolve challenges with transparency.	The QA officer pursues opportunities to demonstrate open communication and cross-team sharing for new research professionals in such a way that colleagues can incorporate them into their practices, for example, the development of standard operating procedures for informed consenting. They provide opportunities for bidirectional feedback to improve openness for their self and their mentees.

**Table 6.** Vignette 2: the quality assurance (QA) officer supports “team learning and adapting behaviors” and implements leveled “change and team growth”

Fundamental	Skilled	Advanced
***Recognize*** *various communication methods and team preferences*	***Exhibit*** *preferred team communication methods*	***Construct*** *team communication methods for process improvement*
The QA officer identifies and considers multiple communication methods that clinical research team members utilize during the informed consent process. They acknowledge team preferences and the necessity of each modality, including using electronic health record systems to maintain patient privacy or clinical trial management systems for digital document storage and centralized access.	With intentionality, the QA officer implements the team’s preferred communication methods to enhance learning opportunities. Each team member is encouraged to practice mutually agreeable methods of communication during the informed consent process. The communication methods are comprehensible to all parties involved.	At mutually agreed-upon intervals, the team uses its preferred methods to reevaluate the style and efficiency of communication styles. Through a shared and diverse methodology, the team analyzes the results of adherence to good clinical practice and clarity of communication through the consent process to identify areas for improvement. The team collaborates to determine quality improvement, implementation, and evaluation of the informed consenting process.

## Discussion

Effective and successful clinical research is highly dependent upon fully functioning teams of diverse professionals spanning multiple disciplines who may be geographically dispersed and connected virtually. Team development has been the subject of early training in teaming, namely the process of forming the team (membership, identity), storming (defining purpose, goals), norming (developing trust, reliance on one another), performing (team tasks) and adjourning (when teams come to an end) [13,36]. However, in the complex clinical research setting, interdisciplinary teams intersect continually in a seemingly three-dimensional space. Therefore, establishing team science competencies and competency training could strengthened the capacity and performance of clinical translational researchers and trainees [37]. A similar need exists for CRPs, the heterogeneous professional staff who operationalize clinical research. Our Delphi study contributes a set of leveled CRP team science competencies (fundamental, skilled, and advanced) that can serve as a basis for future training, role progression, and research. One study related to CRP team science for a pediatric research network that applied the principles of storming, norming, and performing to reach project aims or improve connections across the network [38]. However, the majority of current clinical research team science literature focused on the faculty researchers/principal investigators and trainees, with a paucity of literature on CRPs.

The individual and team competencies of Lotrecchiano *et al.* [23] serve as a basis for this work expanding the 13 competencies to 59 CRP team science smart skills and associated skills examples at the fundamental, skilled, and advanced levels. Included are sample vignettes to illustrate the application of the leveling concepts for potential training. This work may be helpful in improving CRP retention and job satisfaction, which is currently an industry-wide challenge [8]. For example, the leveled team science smart skills could be added to job descriptions and evaluation criteria. It can inform team training to improve team function. Moreover, it can be incorporated into DEIA, soft skills, emotional intelligence, and communication training to better serve diverse teammates and study participants.

The JTF Framework was first published ten years ago, and the competency domains have been updated in response to the evolving clinical research enterprise [39]. For example, the need for project management competencies led to a working group contributing additional leveled competencies in clinical research project management [40]. Moreover, new clinical research competencies are being identified for JTF Domain 6: Data Management and Informatics in response to expanding data management, informatics, and digital health technologies [41]. Moreover, the Association of Clinical Research Professionals (ACRP) and Society of Clinical Research Associates (SoCRA) certifications concentrate on Domains 1 through 6 in their certification review materials and targeted training [4,5]. Within the JTF Framework, Domains 7 (Leadership and Professionalism) and Domain 8 (Communication and Teamwork) have only four core competencies. However, this newly defined set of team science competencies enhances the established JTF competencies by contributing to the robustness of JTF Domains 7 and 8 and brings forward the conversation about CRPs as members of clinical research teams.

A limitation of this work is that it was based on one team science model. However, defining CRP specific skills for existing individual and team competencies provided an intuitive framework to branch out the leveled skills. Moreover, given the length of the project, four of the 18 volunteer members of our team rotated off the group after six months due to competing commitments. Ideally a Delphi group would remain stable throughout the project. Finally, the skills defined by this team are not meant to be exhaustive, but rather provide a foundation from which to build further team science competencies, skills, and training for CRPs, and a framework for future research.

Defining team science competencies contextualized across the career life course, (fundamental, skilled, and advanced), can meet the CRP workforce where they are and contribute to professional development as they progress. By applying the individual and team competency framework selected for this project, we identified 59 smart skills that were leveled across that career progression [23]. This work sets the stage for future educational and research applications. Training CRPs using vignettes, video-scaping, and workshops can be innovative vehicles for CRP staff development. Developing team science skills can strengthen effective working relationships across interdisciplinary clinical research teams and contribute to a stable, more satisfied CRP workforce. Developing team science skills for CRPs may contribute to more effective collaborations across interdisciplinary clinical research teams. These skills may also improve research outcomes and stabilize the CRP workforce.

## Supplementary material

The supplementary material for this article can be found at https://doi.org/10.1017/cts.2024.509.
